# ODF2 Negatively Regulates CP110 Levels at the Centrioles/Basal Bodies to Control the Biogenesis of Primary Cilia

**DOI:** 10.3390/cells12172194

**Published:** 2023-09-01

**Authors:** Madeline Otto, Sigrid Hoyer-Fender

**Affiliations:** Johann-Friedrich-Blumenbach-Institute of Zoology and Anthropology—Developmental Biology, GZMB, Ernst-Caspari-Haus, Justus-von-Liebig-Weg 11, Georg-August-Universität Göttingen, 37077 Göttingen, Germany

**Keywords:** ODF2, CP110, NEURL4, HYLS1, ciliation, rapamycin-induced targeting

## Abstract

Primary cilia are essential sensory organelles that develop when an inhibitory cap consisting of CP110 and other proteins is eliminated. The degradation of CP110 by the ubiquitin-dependent proteasome pathway mediated by NEURL4 and HYLS1 removes the inhibitory cap. Here, we investigated the suitability of rapamycin-mediated dimerization for centriolar recruitment and asked whether the induced recruitment of NEURL4 or HYLS1 to the centriole promotes primary cilia development and CP110 degradation. We used rapamycin-mediated dimerization with ODF2 to induce their targeted recruitment to the centriole. We found decreased CP110 levels in the transfected cells, but independent of rapamycin-mediated dimerization. By knocking down ODF2, we showed that ODF2 controls CP110 levels. The overexpression of ODF2 is not sufficient to promote the formation of primary cilia, but the overexpression of NEURL4 or HYLS1 is. The co-expression of ODF2 and HYLS1 resulted in the formation of tube-like structures, indicating an interaction. Thus, ODF2 controls primary cilia formation by negatively regulating the concentration of CP110 levels. Our data suggest that ODF2 most likely acts as a scaffold for the binding of proteins such as NEURL4 or HYLS1 to mediate CP110 degradation.

## 1. Introduction

Primary cilia are ubiquitous sensory organelles, essential for the transmission of mechanical and chemical signals toward the cell center and are thus crucial for embryonic and postnatal development and tissue homeostasis [1,2,3,4,5,6,7,8]. The assembly and disassembly of primary cilia in cycling cells correlate with cell cycle progression, and they are predominant in cell cycle arrested, quiescent cells [9,10,11,12,13,14,15,16].

The primary cilium is an outgrowth of the basal body that extends from its distal region to eventually project into the cellular environment, while the basal body anchors the cilium inside the cell and to the cell membrane [17,18]. The basal body itself is a derivative of the older or mother centriole of the pair of centrioles that form the centrosome, which is the main microtubule-organizing center (MTOC) of the cell [19]. Both centrioles of the centrosome—the mother centriole and its descendant, the daughter centriole—differ in age, structure, protein composition, and function. The mother centriole is characterized by the presence of distal (DA) and subdistal (SDA) appendages, and it is the mother centriole that nucleates and anchors microtubules (MTs) and assembles a ciliary axoneme. The initiation of cilium assembly starts with the docking of vesicles at the distal end of the mother centriole, which, additionally, marks the transformation of the mother centriole into the basal body, a process that must be precisely regulated. In addition to the hundreds of proteins required to build and maintain primary cilia, the onset of cilia formation also requires the reorganization and remodeling of the mother centriole [17,20,21,22].

At first, after the initiation of centriole duplication, a specific set of proteins is recruited to the nascent daughter centriole. These daughter centriole-enriched proteins (DCPs) include CEP120, centrobin, and NEURL4, which are also recruited in this order [23,24,25]. DCPs are removed in the next cell cycle, highlighting the conversion of the daughter centriole into a mature centriole and the formation of its characterizing DAs and SDAs. DAs and SDAs are assembled by the sequential recruitment of specific proteins [26,27]. The protein CEP164, which is involved in the first step in cilia formation, namely the docking of ciliary vesicles, is located at the tip of DAs [28,29]. The assembly of SDAs is centered around ODF2, which is located near the centriole wall [30].

Immediately after ciliary vesicle docking, or concurrent with it, the ciliary axoneme is formed by extension from the distal end of the mother centriole/basal body [31]. Axoneme extension requires the elimination of the centriolar coiled coil protein of 110 kDa, in short CP110, and its interacting partner CEP97 from the distal end of the mother centriole [16,32,33]. CP110 and its associated partners form a cap at the distal ends of the two centrioles, which prevents microtubule elongation and axoneme extension [34,35]. In contrast to its suppressive role in cilia formation in cultured cells, CP110 is also required for SDA assembly and cilia formation in vivo [36,37]. Its dual role as a suppressor and promoter of ciliogenesis suggests that its optimal cellular level needs to be balanced [38].

CP110 levels are mainly regulated via the ubiquitin-dependent proteasome pathway [39,40]. Ubiquitylation and destabilization of CP110 are promoted by its interactor NEURL4, a protein preferentially located at procentrioles and daughter centrioles [25]. Early during ciliogenesis, in a process that requires mother-daughter centriole proximity, NEURL4 translocates to the mother centriole, which is necessary for the degradation of CP110 and cilia formation [41]. NEURL4, which has no ubiquitin ligase activity, is a substrate of the HECT-E3 ligase HERC2, and both are found in a complex containing CP110. Thus, the NEURL4-HERC2 complex seems to be responsible for the regulation of CP110 degradation [42]. The targeting of NEURL4 to the centrosome was shown to be sufficient to remove CP110 and restore ciliogenesis [41]. The removal of the CP110-CEP97 complex then allows the extension of microtubules to form the ciliary axoneme.

Furthermore, additional proteins and CP110 binding partners are involved in the regulation of cilium assembly [32]. The removal of CP110 and ciliogenesis requires the serine/threonine kinase TTBK2 [43,44,45,46]. TTBK2 binds to CEP164 at the DAs, which is regulated by the phosphatidylinositol(4)P 5-kinase (PIPKIγ)-mediated depletion of phosphatidylinositol-4-phosphate (PtdIns(4)P) levels at the centrosome/ciliary base [47,48]. PIPKIγ, furthermore, interacts and recruits HYLS1 (hydrolethalus syndrome protein 1) to the ciliary base, causing the activation of PIPKIγ and in turn, the accelerated depletion of centrosomal PI(4)P and axoneme extension [49,50]. In addition to TTBK2, the microtubule (MT)-associated protein/MT affinity regulating kinase 4 (MARK4) is required for the initiation of axoneme extension [51]. MARK4 depletion prevented the exclusion of CP110 from the mother centriole and blocked ciliogenesis.

MARK4 is associated with the basal body and the ciliary axoneme and interacts with and phosphorylates ODF2 (alias cenexin), the centriole and sub-distal appendage protein that is mandatory for primary cilia formation and viability [52]. *Odf2*-deficient mice die during preimplantation [53]. *Odf2*-deficient centrioles in mouse embryonic carcinoma F9 cells lack appendages and are unable to generate a primary cilium [54]. Furthermore, a crucial amount of ODF2 at the centrosome/basal body is necessary for the initiation of ciliogenesis [55]. ODF2 specifically interacts with the GTPase membrane trafficking regulator Rab8a that facilitates the docking of ciliary vesicles to DAs and the formation of the ciliary membrane [56,57].

Here, we investigated the suitability of the rapamycin-induced dimerization system for centriolar targeting and asked whether the recruitment of NEURL4 or HYLS1 to centrioles affects cilia formation and CP110 levels. Rapamycin induced dimerization by binding to FKBP12, which contains the FK506 binding protein, and then to the FKBP and rapamycin binding domain (FRB) of mTOR, forming a ternary complex [58]. FKBP12 and FRB were fused to either ODF2 or NEURL4 and HYLS1, respectively. We found reduced CP110 levels in transfected cells that were independent of NEURL4 recruitment, indicating that ODF2 is responsible for CP110 removal. We then investigated the interrelationship between ODF2 and CP110 and demonstrated that CP110 levels at the centriole and the basal body are controlled by ODF2. Finally, we asked whether primary cilia formation is promoted by overexpression of either ODF2, NEURL4, or HYLS1, or the ODF2-mediated centriolar recruitment of NEURL4 or HYLS1. Our data show that although ODF2 controls CP110 levels at the centrosomes and basal bodies, primary ciliation was largely unaffected. In contrast, we observed a significant increase in ciliated cells by overexpression of either NEURL4 or HYLS1. The overexpression of both ODF2 and HYLS1 initiated the formation of intracytoplasmic tubes, suggesting the interaction between both proteins that support the formation and/or stabilization of higher-order structures. Our data show that ODF2 is essential for the removal of CP110 and suggest that CP110 levels must be strictly controlled to allow for cilium formation.

## 2. Materials and Methods

### 2.1. Plasmid Constructs

For targeted recruitment, the coding sequence of *Odf2*, subclone *138NC*, was ligated in-frame to *mRFP-FKBP* at its 3′-end in the vector backbone of *pEGFP-N1*, which was deleted for the coding sequence of *egfp*, yielding plasmid *p138NC(Odf2)::mRFP::FKBP*. The 138NC targets the centrosome and lacks 173aa at its C-terminal end, otherwise present in human cenexin (Q5BJF6.1) [59]. *Neurl4* was in-frame ligated to FRB-ECFP at its N-terminal end in plasmid *pCR3.1,* yielding plasmid *pFRB::ECFP::Neurl4*. The full-length coding sequence of 962bp of *Hyls1* was isolated from mouse testis cDNA by RT-PCR using primer pair Hyls_NotI_rev (agcggccgcttaagaaggagaaagcgg)/Hyls1_BglII_for (cagatctatggcagaaaaaagacaagc) and N-terminally ligated to FRB::ECFP in *pCR3.1,* yielding plasmid p*FRB::ECFP::Hyls1*. Centrin-2 fused to cherry (*pCentrin-2::Cherry*) was transfected for the identification of centrosomes by auto-fluorescence. Clones were sequenced to verify the correct open reading frame.

### 2.2. Immune-Cytology and Protein Quantification

NIH3T3 cells were obtained from DSMZ (ACC59) and cultivated in Dulbecco’s Modified Eagle’s Medium (DMEM; GlutaMax^TM^ with high glucose concentration (4.5 g/L); #10566, Thermo Fisher Scientific, Waltham, MA, USA), supplemented with 1000 U/mL penicillin and 1000 µg/mL streptomycin and either 10% (*v*/*v*) fetal calf serum (FCS) (normal medium, NM) or 0.5% FCS (serum starvation medium, SSM). Cells were cultivated at 37 °C and 5% CO_2_. The cultivation in serum starvation medium was performed for 24 h or 48 h.

For immune-cytology, cells were seeded at a density of 2 × 10^5^ cells per well of a 6-well plate on glass coverslips and fixed in 3.7% paraformaldehyde (PFA) for 20 min at 4 °C. The cells were then permeabilized with 0.3% Triton X-100 in PBS (phosphate-buffered saline) for 10 min at room temperature and blocked by incubation in PBS containing 1% bovine serum albumin (BSA) and 0.5% Tween-20 for at least 1 h. The samples were incubated with the primary antibodies anti-acetylated α-tubulin (clone 6-11B-1; Santa Cruz Biotechnology, Inc., Heidelberg, Germany, #sc-23950, diluted 1:50), anti-ARL13B (#17711-1-AP, diluted 1:400, Proteintech, St. Leon-Rot, Germany), anti-ODF2 (ESAP15572, diluted 1:100, antibodies-online GmbH, Aachen, Germany), anti-CP110 (#12780-1-AP, diluted 1:100, Proteintech, St. Leon-Rot, Germany), or anti-GFP (either monoclonal mouse IgG2a (3E6), A-11120, lot #7101-1, Molecular Probes, Inc., Eugene, OR, USA, or polyclonal rabbit anti-GFP, self-made) at 4 °C overnight. The secondary antibodies used were goat anti-mouse-IgG-DyLight 488 (#35503, diluted 1:300, Thermo Fisher Scientific, Waltham, MA, USA), goat anti-rabbit-MFP590 (#MFP-A1037, diluted 1:100, Mobitec, Göttingen, Germany), goat anti-rabbit-IgG-Dylight 488 (#35553, Thermo Fisher Scientific, Waltham, MA, USA), and goat anti-rabbit-AlexaFluor-647 (#A-21245, lot AB_2535813, Thermo Fisher Scientific, Waltham, USA) or goat anti-mouse-AlexaFluor-647 (#A-21235, lot AB_2535804, Thermo Fisher Scientific, Waltham, MA, USA). DNA was counterstained with DAPI (4′, 6-diamidino-2-phenylindole; Vector Lab., cat. no. H-1500). Images were captured by confocal microscopy (LSM 980, Carl Zeiss AG, Oberkochen, Germany) and processed using Adobe Photoshop 7.0. For protein quantification, all pictures were captured using identical settings, and intensities were measured using Fiji [60]. The relative quantity of the protein in question was obtained by subtraction of the average intensity of the centrosomal/basal body background, always using four different areas for each quantification, from the intensity of the protein in question, followed by dividing the intensity through the area. Fold changes were calculated by relating the relative quantities to the average relative quantity of the control. The diameter of ODF2/HYLS1 structures was measured with Fiji. The counting of primary cilia was performed by visual inspection and scanning through all focal planes.

### 2.3. Transfection of Cells

NIH3T3 cells were seeded at a density of 1.25 × 10^5^ on coverslips in 6-well plates. Transfection was conducted 24 h post-seeding, and cells were processed for immune-cytology 24 h later. Plasmid DNA or siRNA were transfected using EndoFectin^TM^ Max Transfection Reagent, following the manufacturer’s instructions (#EF014, GeneCopoeia, Inc., Rockville, MD, USA). For *Odf2* knockdown, the short hairpin constructs *sh3* (specifically targeting sequence gaactcctccaggagatac of mouse *Odf2/cenexin*; [61] or *Odf2* siRNA (stealth siRNA ODF2MSS207236; final concentration 40 nM; Life Technologies, Carlsbad, CA, USA) were used. As controls, the plasmid *K07* (OriGene Technologies, Inc., Rockville, MD, USA), which lacked homology with any known mRNA, or the control siRNA (siGenome Non-targeting siRNA #1; D-001210-01-05; target sequence UAGCGACUAAACACAUCAA; Dharmacon, Lafayette, CO, USA) were used. Additionally, for rescue, the expression plasmid encoding human cenexin (*hCenexin*) [62] was co-transfected. The co-transfection of *pCentrin-2::Cherry* was used to identify the centrosome in the transfected cells.

### 2.4. Rapamycin-Induced Dimerization

Cells were transfected with *p138NC::mRFP::FKBP* and either *pFRB::ECFP::Neurl4* or *pFRB::ECFP::Hyls1,* and dimerization was induced by incubation with rapamycin (#553210, 1 µM stock solution in DMSO, Millipore, Burlington, MA, USA). As a control, the cells were incubated with the same amount of DMSO, which was used as the solvent for rapamycin, for the indicated time. For quantification of CP110 at the basal bodies, the cells were incubated with anti-CP110 antibodies (#12780-1-AP, diluted 1:100, Proteintech, St. Leon-Rot, Germany), followed by goat anti-rabbit-IgG-AlexaFluor-647 (#A-21245, lot AB_2535813, Thermo Fisher Scientific, Waltham, MA, USA). The basal bodies were identified by the decoration of the primary cilium with anti-acetylated tubulin antibodies, followed by goat anti-mouse-IgG-Dylight 488 (#35503, Thermo Fisher Scientific, Waltham, MA, USA).

## 3. Results

### 3.1. ODF2-Mediated Recruitment of NEURL4 to the Centrosome

**Figure 1:**
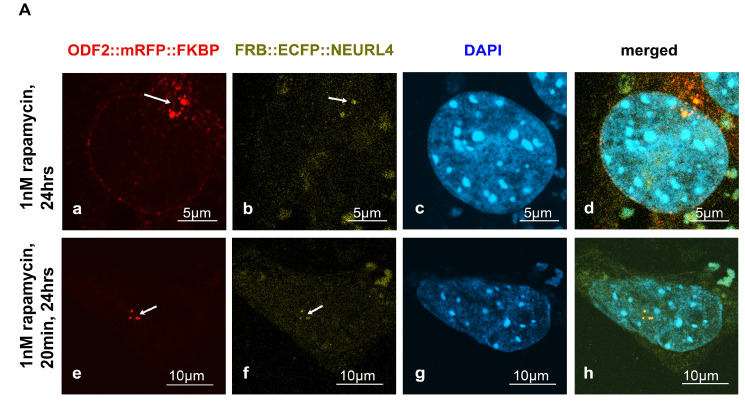

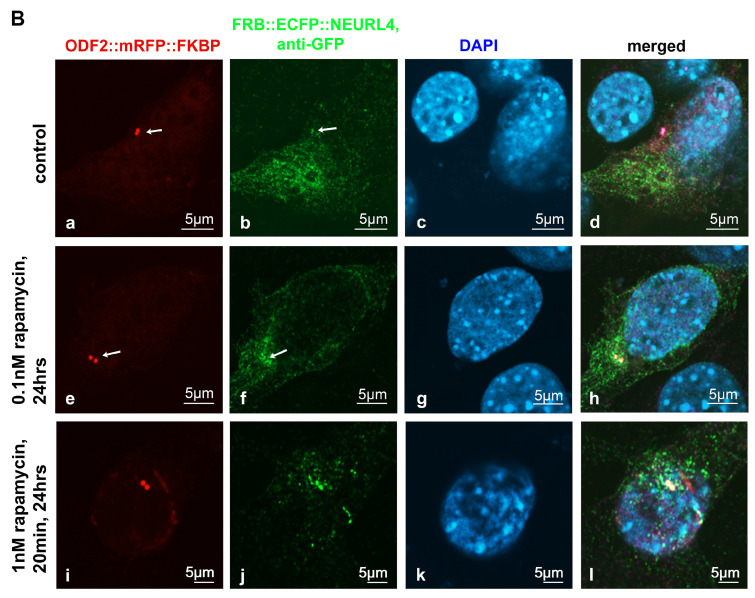
Induced recruitment of NEURL4 to the centrosome by rapamycin-mediated dimerization with ODF2. NIH3T3 cells were transfected with plasmids encoding ODF2 (138NC; 138NC::mRFP::FKBP) and FRB::ECFP::NEURL4. (**A**) Centrosomal recruitment of FRB::ECFP::NEURL4 detected by autofluorescence of either mRFP ((**a**,**e**), in red) or ECFP ((**b**,**f**), in green). Nuclear staining with DAPI ((**c**,**g**), in blue) and merged images (**d**,**h**). Scale bars of 5 µm (**a**–**d**) or 10 µm (**e**–**h**). Incubation with rapamycin for either 24 h (1 nM rapamycin, (**a**–**d**)) or 20 min (1 nM rapamycin), followed by medium exchange and incubation without rapamycin for 24 h (**e**–**h**). (**B**) Decoration of FRB::ECFP::NEURL4 with anti-GFP antibodies. Autofluorescence of the mRFP-tag of ODF2 ((**a**,**e**,**i**); in red) at the centrosome (arrows). NEURL4 was immunologically detected with anti-GFP ((**b**,**f**,**j**), in green). Nuclear staining with DAPI ((**c**,**g**,**k**), in blue) and merged images (**d**,**h**,**l**). Scale bars are 5 µm. Incubation with 2 µL of DMSO for 24 h (**a**–**d**) as the control. Incubation with rapamycin for either 24 h (0.1 nM rapamycin, (**e**–**h**)) or 20 min (1 nM rapamycin) followed by medium exchange and incubation without rapamycin for 24 h (**i**–**l**).

The removal of the CP110 protein module from the distal end of the mother centriole is essential for axoneme extension and primary cilia formation. The degradation of CP110 is mainly regulated by the ubiquitin-dependent proteasome pathway, in which NEURL4 plays an essential role. We asked whether targeted recruitment of NEURL4 to the distal end of the centrioles is sufficient to induce CP110 removal and in turn, to promote axoneme extension. We used the rapamycin system to chemically induce the recruitment of NEURL4 by the transfection of plasmids *p138NC(Odf2)::mRFP::FKBP* and *pFRB::ECFP::Neurl4.* Rapamycin binds to both, FKBP and FRB, and thus caused their dimerization. The 138NC/ODF2 is intrinsically located in the centrosome and thus recruits NEURL4 to the centrosome by rapamycin-induced dimerization. ODF2 was detected by the autofluorescence of the mRFP-tag. Although recruitment of FRB::ECFP::NEURL4 by rapamycin was already visible by the ECFP autofluorescence (Figure 1A(b,f)), the ECFP fluorescence was weak. Therefore, we enhanced the fluorescence of FRB::ECFP::NEURL4 by decoration with anti-GFP antibody (Figure 1B). We found very weak staining of the centrosome for FRB::ECFP::NEURL4 in the control cells, which have been incubated with DMSO for 24 h (Figure 1B(a–d)), but a strong increase at the centrosome by the rapamycin-induced dimerization with 138NC(ODF2)::mRFP::FKBP (Figure 1B). Incubation with 0.1 nM of rapamycin for 24 h caused the centrosomal recruitment of FRB::ECFP::NEURL4 (Figure 1B(e–h)), which is much stronger when ten times the amount of rapamycin was used, but only for 20 min, followed by medium exchange and incubation of the cells in standard medium without rapamycin (Figure 1B(i–l)).

### 3.2. Rapamycin-Induced Recruitment of NEURL4 to the Centrosome Did Not Enhance the Loss of CP110

**Figure 2:**
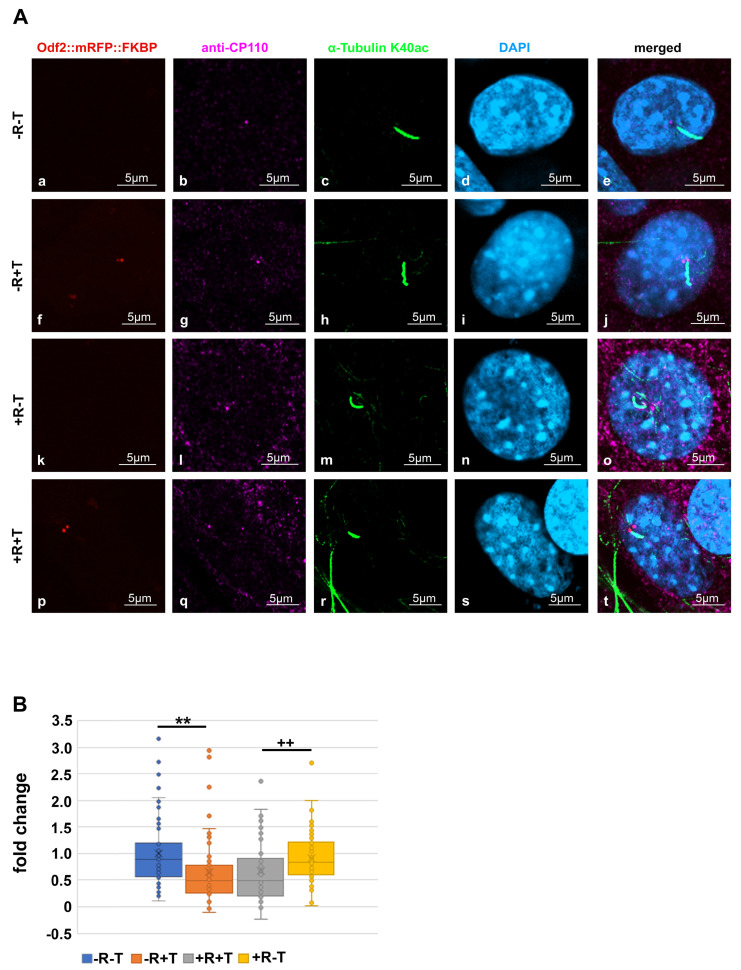
Increased amount of ODF2 at the basal body, but not the rapamycin-induced recruitment of NEURL4, caused a loss of CP110. NIH3T3 cells were transfected with *p138NC/Odf2::mRFP::FKBP* and *pFRB::ECFP:::Neurl4* (+T) and incubated, either with (+R) or without rapamycin (−R). Non-transfected cells (−T) served as controls. (**A**) Detection of the basal body in transfected cells by autofluorescence of the mRFP-tagged ODF2 ((**a**,**f**,**k**,**p**); in red). Anti-CP110 staining (**b**,**g**,**l**,**q**) (in magenta). Basal bodies were identified by their association with the ciliary axoneme decorated with acetylated tubulin ((**c**,**h**,**m**,**r**); in green). DAPI staining of nuclei ((**d**,**i**,**n**,**s**); in blue) and merged images (**e**,**j**,**o**,**t**). Scale bars are 5 µm. (**B**) CP110 was identified by antibody staining, and the CP110 area was captured by confocal imaging. CP110 intensities of individual basal bodies were quantified, followed by background subtraction, which was calculated as the average intensity of four different neighboring areas, and the corrected CP110 intensity was divided through the area. The average of the calculated CP110 intensities of the basal bodies of control cells (non-transfected and not incubated with rapamycin, −R−T) served as the reference to which all calculated CP110 intensities were related, giving the fold changes. In transfected cells, the average CP110 intensities were 0.658× (−R+T) and 0.6629× (+R+T) compared to the control (−R−T, 1×), whereas rapamycin-incubation in non-transfected cells (+R-T) had no effect, showing an average intensity of 0.9124×. A significant decrease in CP110 was found in the transfected cells compared to the non-transfected cells, both in the absence of rapamycin (−R+T compared to −R−T; *p* = 0.001472 **) and in the presence of rapamycin (+R+T compared to +R−T; *p* = 0.005132 **^++^**). (Student’s *t*-test, two-tailed, homoscedastic). *N* individual quantifications were performed: *n* = 92 (−R−T), *n* = 54 (−R+T), *n* = 88 (+R−T), *n* = 50 (+R+T). *p* < 0.01 ** and **^++^**.

Since NEURL4 plays an essential role in CP110 removal, we next investigated whether the induced recruitment of NEURL4 to the basal bodies affected the amount of CP110. NIH3T3 cells were transfected with *p138NC/Odf2::mRFP::FKBP* and *pFRB::ECFP::Neurl4*, and their dimerization was induced by incubation with rapamycin. To identify basal bodies, the primary cilium was highlighted by acetylated tubulin staining (Figure 2A). CP110 was decorated by antibody staining and quantified. Transfected cells without rapamycin incubation and non-transfected cells with or without rapamycin incubation served as controls. We found a significant decrease in CP110 in transfected cells (+T) compared to non-transfected cells (T) in both cases, either without rapamycin-incubation (−R; *p* = 0.001472 **) or with rapamycin-induced NEURL4 recruitment (+R; *p* = 0.005132 **). No effect on the amount of CP110 was found from the rapamycin-incubation of either non-transfected cells (+R−T compared to −R−T; *p* = 0.2759) or transfected cells (+R+T compared to −R+T; *p* = 0.968234) (Figure 2B). Thus, our data show that the ODF2-mediated, rapamycin-induced recruitment of NEURL4 to the basal body did not cause a decrease in CP110. Furthermore, the data indicate that ODF2 is responsible for the loss of CP110.

### 3.3. Distribution of ODF2 and CP110 at the Centrosome and the Basal Body

**Figure 3:**
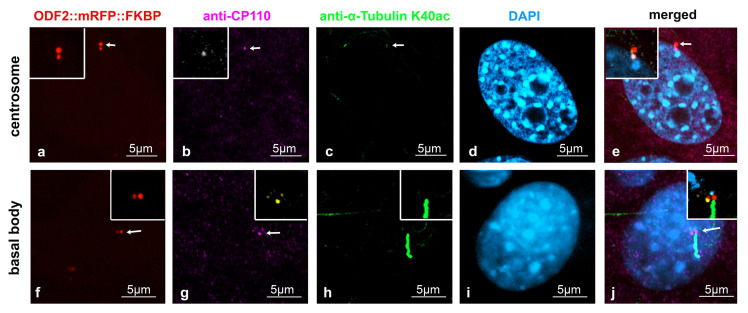
ODF2 and CP110 levels are inversely correlated at centrosomes and basal bodies. NIH3T3 cells were forced to express ODF2 fused to mRFP (138NC/ODF2::mRFP::FKBP) ((**a**,**f**), red), which was detected by the autofluorescence of mRFP, whereas CP110 (anti-CP110, (**b**,**g**), pink; inset (**b**), white, and (**g**), yellow) and acetylated tubulin (anti-α-tubulin K40ac, (**c**,**h**), green) were detected by antibody staining. The centrosome ((**c**), arrow) and the primary cilium at the basal body (**h**) were decorated by acetylated tubulin. Nuclei were stained with DAPI ((**d**,**i**), blue). Merged images in (**e**,**j**). Scale bars are 5 µm. Arrows highlight centrosomes and basal bodies. The insets show the magnified areas of the centrosome and basal body, with CP110 stained from pink to white (**b**,**e**) or to yellow (**g**,**j**) to better distinguish between ODF2 and CP110 in the merged images.

Our data indicated that the removal of CP110 is controlled by ODF2. Therefore, we looked first at the distribution of both ODF2 and CP110 in centrosomes and basal bodies in the NIH3T3 cells. Decoration of the cilium by anti-acetylated tubulin staining served to discriminate between the centrosomes and basal bodies. To allow for the concurrent detection of ODF2 and CP110, the cells were transfected with a plasmid encoding ODF2 fused to mRFP (*p138NC::mRFP::FKBP*) for the autofluorescence-detection of ODF2 and immunologically stained for CP110 and acetylated tubulin. ODF2 predominantly labeled the mature centriole in the centrosome and the basal body, which is located at the base of the primary cilium, whereas the associated daughter centriole, although decorated with ODF2, showed a much weaker staining (Figure 3a,f). CP110 essentially decorates only one of the two centrioles (Figure 3b,g). Thus, an inverse relationship between the amount of both proteins seems to exist, in that a large amount of ODF2 is associated with low CP110 levels and vice versa, corroborating our previous data.

### 3.4. ODF2 Promotes Depletion of Centrosomal CP110

**Figure 4:**
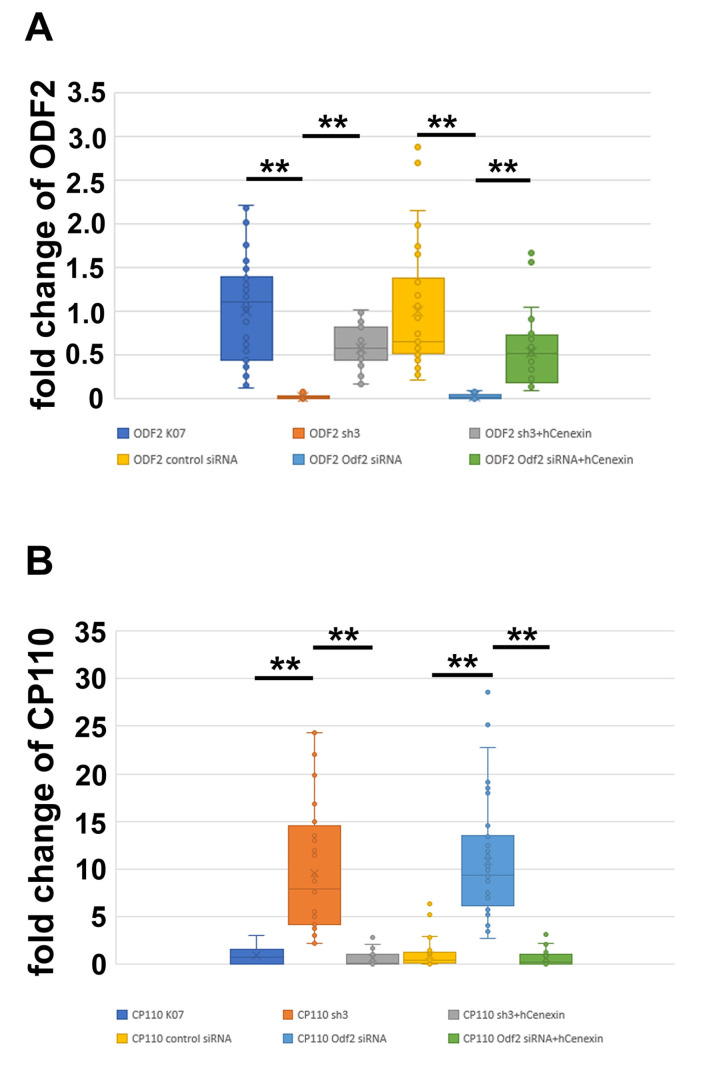
Inverse correlation between the amount of ODF2 and CP110 at the centrosome. ODF2 was depleted by the transfection of either the short hairpin plasmid *sh3* or siRNA. Depletion was rescued by co-transfection of *hCenexin* (*sh3* + *hCenexin* or *Odf2* siRNA + *hCenexin*). *K07* or control siRNA served as the controls. (**A**) Centrosomal quantification of ODF2 revealed significant ODF2-depletion by either *sh3* or *Odf2* siRNA transfection, and a successful rescue by co-transfection of *hCenexin*. Three biological replicates were used for analyses with *n* individual centrosomes: *K07 n* = 48, *sh3 n* = 19, *sh3* + *hCenexin n* = 35, control siRNA *n* = 35, *Odf2* siRNA *n* = 29, *Odf2* siRNA + *hCenexin n* = 31. One-way ANOVA with the Tukey HSD post-hoc test (*p* < 0.01 **). (**B**) Quantification of CP110 at the centrosome in ODF2-depleted cells. Three biological replicates were used for analyses with *n* individual measurements: *K07 n* = 17, *sh3 n* = 44, *sh3* + *hCenexin n* = 22, control siRNA *n* = 33, *Odf2* siRNA *n* = 40, *Odf2* siRNA + *hCenexin n* = 28. One-way ANOVA with the Tukey HSD post-hoc test (*p* < 0.01 **).

To investigate whether the amount of CP110 is affected by ODF2, we modified the amount of ODF2 by either depletion or overexpression and quantified CP110 at the centrosome. The cells were transfected with *pCentrin::Cherry* for decoration and identification of the centrosome, and ODF2 was depleted by the co-transfection of either a short hairpin plasmid targeting *Odf2* (sh3) or *Odf2* siRNA. For rescue, *hCenexin* was co-transfected. As controls, either the non-targeting control plasmid (K07) or a scrambled control siRNA were transfected. Successful depletion or rescue of ODF2 was verified by the quantification of ODF2 at the mother centriole (Figure 4A). We found a reduction in ODF2 by *sh3*-mediated depletion to ~0.03-fold compared to the control plasmid *K07*, and an increase by co-transfection in *sh3* + *hCenexin* to ~0.6-fold of the control. Depletion of ODF2 by *Odf2* siRNA caused a reduction to ~0.03-fold compared to the scrambled control siRNA and an increase to ~0.53-fold compared to the scrambled control siRNA in the rescue experiment. Thus, ODF2 was significantly depleted by both, either the short hairpin plasmid *sh3* or by *Odf2* siRNA, compared to the respective controls, either *K07* or scrambled siRNA. Additionally, co-transfection of *hCenexin*-plasmid with either the *sh3* plasmid or the *Odf2* siRNA rescued the respective depletion (*sh3* + *hCenexin* to *sh3*, *Odf2* siRNA + *hCenexin* to *Odf2* siRNA). The one-way ANOVA with the Tukey HSD post-hoc test revealed significant differences (*p* < 0.01 **).

We then quantified CP110 at the centrosome in cells either depleted or rescued for ODF2 (Figure 4B). As before, the centrosome was identified by Centrin::Cherry fluorescence. We found an increase in centrosomal CP110 of ~10× in ODF2-depleted centrosomes by either *sh3* or *Odf2* siRNA transfection compared to the respective controls, either *K07* or control siRNA transfection. The rescue experiments caused an ~0.6× amount of CP110 in the *sh3* + *hCenexin* transfected centrosomes and an ~0.7-fold increase in the *Odf2* siRNA + *hCenexin* transfected cells when compared to the respective controls, either *K07* or control siRNA. The one-way ANOVA with Tukey HSD post-hoc test revealed significant differences (*p* < 0.01 **). Our data show that ODF2 depletion correlated with an increase in CP110 at the centrosome.

**Figure 5:**
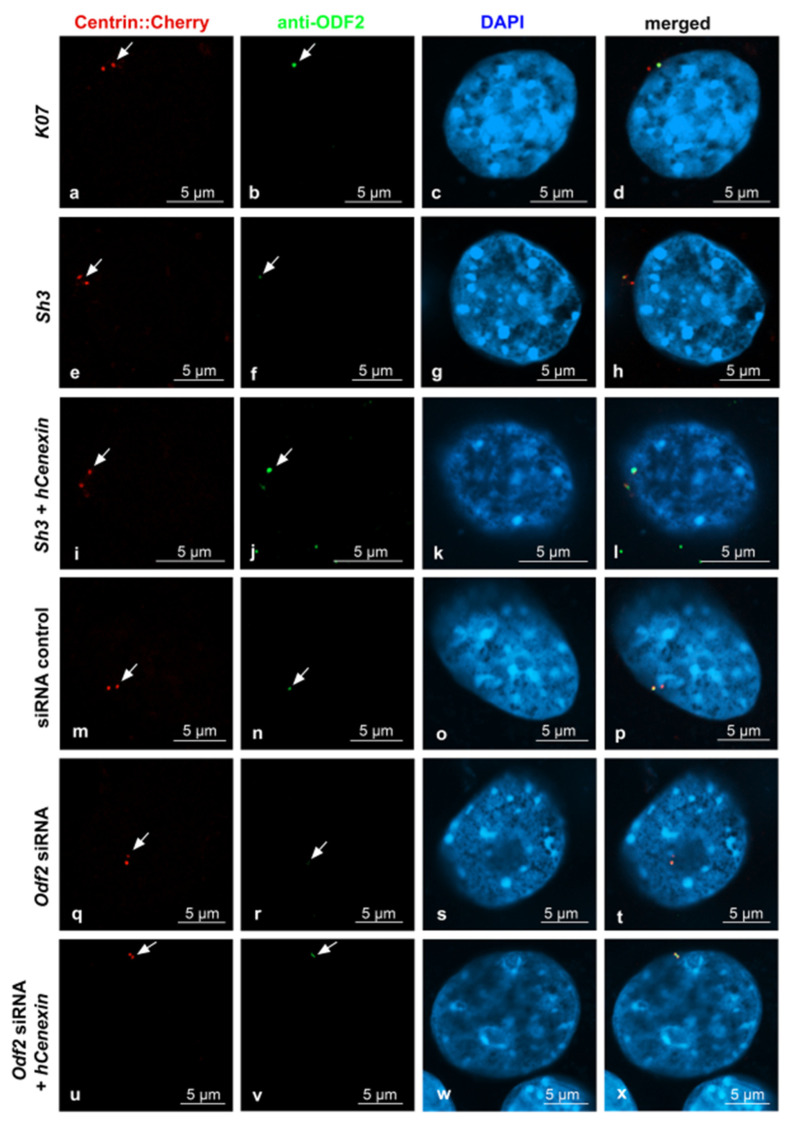
Decrease in centrosomal ODF2 by both, either *sh3*- or *Odf2* siRNA-mediated knockdown. Transfected cells were identified by the autofluorescence of Centrin-Cherry-tagged centrosomes ((**a**,**e**,**i**,**m**,**q**,**u**), in red). ODF2 was detected by antibody staining ((**b**,**f**,**j**,**n**,**r**,**v**), in green). Nuclear stain with DAPI ((**c**,**g**,**k**,**o**,**s**,**w**), in blue). Merged images in (**d**,**h**,**l**,**p**,**t**,**x**). Cells were transfected with either the control plasmid *K07*, the short hairpin plasmid *sh3*, or both *sh3* + *hCenexin* for rescue. Additionally, the knockdown of ODF2 was also achieved by the transfection of *Odf2* siRNA and rescued by the co-transfection of *Odf2* siRNA and *hCenexin*. Control siRNA transfection served as the control experiment. Scale bars are 5 µm. Arrows highlight the centrosomes.

**Figure 6:**
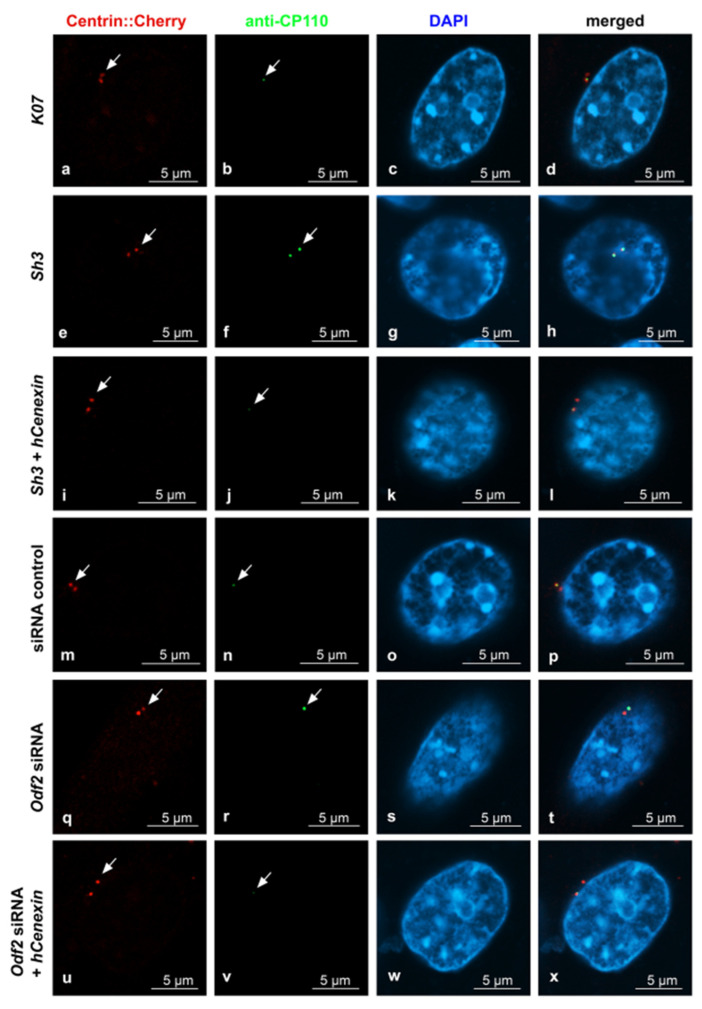
ODF2 knockdown correlated with increased CP110 at the centrosome. Detection of transfected cells by the autofluorescence of Centrin-Cherry-tagged centrosomes ((**a**,**e**,**i**,**m**,**q**,**u**), in red). CP110 was detected by antibody staining ((**b**,**f**,**j**,**n**,**r**,**v**), in green). Nuclear staining with DAPI ((**c**,**g**,**k**,**o**,**s**,**w**), in blue). Merged images in (**d**,**h**,**l**,**p**,**t**,**x**). For ODF2 knockdown, the cells were transfected with the short hairpin plasmid *sh3*, and for rescue, with both *sh3* + *hCenexin*. Additionally, the knockdown of ODF2 was also achieved by transfection of *Odf2* siRNA and rescued by the co-transfection of *Odf2* siRNA + *hCenexin*. Transfection of the control plasmid *K07* or control siRNA served as the reference experiments. Scale bars are 5 µm. Arrows highlight the centrosomes.

Sample pictures used for the quantification of ODF2 and CP110 by ODF2 knockdown are shown in Figure 5 and Figure 6. All pictures were captured using identical settings, and no further processing was performed to allow for both a visual estimation, as well as a correct protein quantification. Centrosomes were identified by Centrin-Cherry auto-fluorescence. Protein quantification of Centrin-Cherry-labeled centrosomes only also ensured that only the transfected cells were included in the analyses. A marked decrease in ODF2 at the centrosome is observed by *sh3*- or siRNA-mediated knockdowns (Figure 5b,f,n,r), as is the rescue of ODF2 by co-transfection of *hCenexin* (Figure 5f,j,r,v). The ODF2 knockdown correlated with increased CP110 at the centrosome (Figure 6b,f,n,r).

### 3.5. Enrichment of ODF2 at the Basal Body

**Figure 7:**
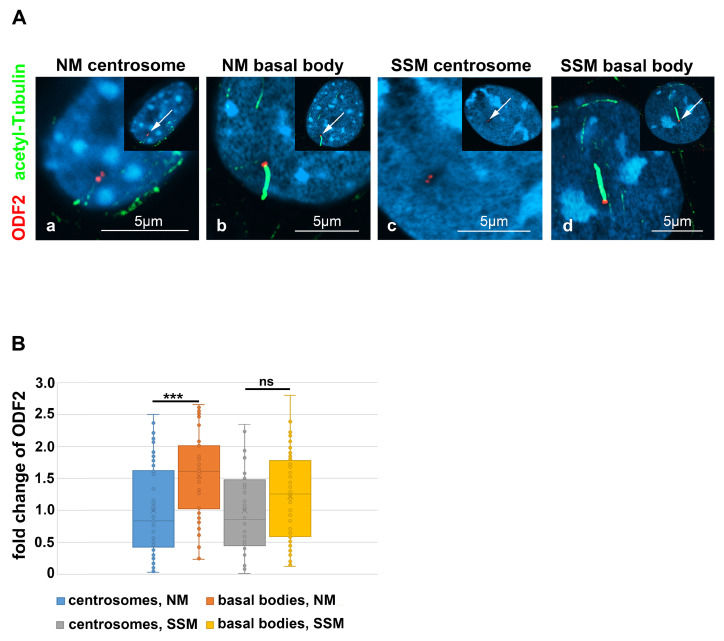
ODF2 is enriched in basal bodies. (**A**) Detection of endogenous ODF2 (in red) in centrosomes and basal bodies of NIH3T3 cells. Cells were cultivated in either normal medium (NM) or serum starvation medium (SSM), and centrosomes (**a**,**c**) and basal bodies (**b**,**d**) were detected by anti-ODF2 (in red) and anti-acetylated tubulin (in green) decoration. Basal bodies were identified by the presence of the primary cilium, which was decorated by anti-acetylated tubulin staining (in green). Nuclear staining with DAPI. All merged images. The insets show an overview of the whole cell, with the enlarged area highlighted by an arrow. Bars are 5 µm. (**B**) ODF2 was enriched in the basal bodies as compared to the centrosomes under both cultivation conditions, either in normal medium or serum starvation medium (*p* < 0.001 ***, not significant ns). Quantification was performed by Fiji. For each quantification, the average intensity of the centrosomal/basal body background (always using four different areas) was subtracted from the intensity of ODF2 staining and the corrected intensity related to the measured area, thus yielding the relative quantity of ODF2. For the fold change calculation, the relative quantities of ODF2 were related to the average relative quantity in the centrosomes, either in NM or SSM, respectively. Three biological replicates with *n* measurements: centrosome in NM *n* = 52, basal body in NM: *n* = 48, centrosome in SSM *n* = 44, basal body in SSM *n* = 66.

ODF2/cenexin is a marker protein for the mature centriole and the basal body and is mandatory for primary cilia formation. Initiation of cilia formation requires the transformation of the mature centriole into a basal body, a process including structural and compositional remodeling, which might also encompass ODF2. We, therefore, asked whether the formation of cilia correlated with an increase in ODF2 and thus quantified the amount of ODF2 in the centrosomes and basal bodies in both the cycling cells and the serum-starved cells that were cultivated in either standard medium (NM) or serum-depleted medium (SSM), respectively. The discrimination between the centrosomes and basal bodies was achieved by the decoration of the axoneme of the primary cilium for tubulin acetylation (Figure 7A, in green). The centrosomes were identified by the detection of ODF2, located in both centrioles, and represented as twin dots, but without an attached axoneme (Figure 7A, in red). We found a significant increase in the ODF2 concentration up to ~1.5-fold in basal bodies compared to the centrosomes in the proliferating cells cultivated in standard medium with 10% FCS (*p* = 0.0001132 ***) (Figure 7B). In the serum-starved cells, the enrichment of ODF2 in basal bodies is ~1.2-fold compared to that of the centrosomes, which is, however, not significant (*p* = 0.08373), according to the two-tailed, homoscedastic Student’s *t*-test. The enrichment of ODF2 thus correlated with the transformation of the centrosome into the basal body, as well as cilium formation.

### 3.6. The overexpression of ODF2 Did Not Promote Cilia Formation

**Figure 8:**
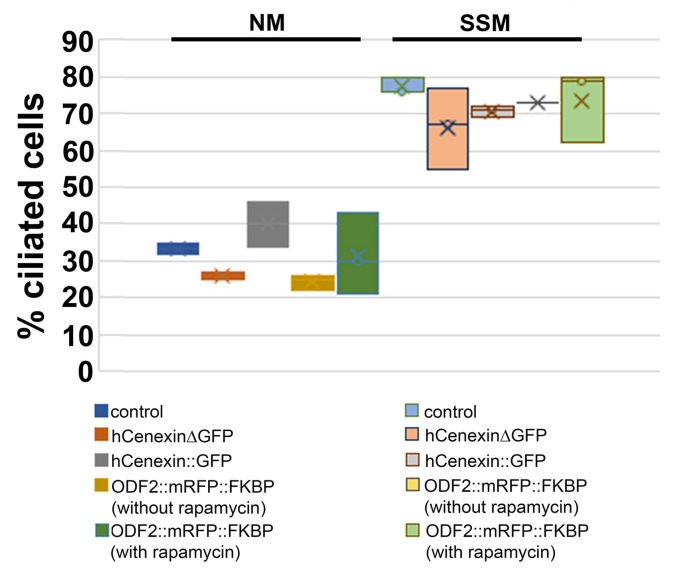
ODF2 overexpression did not promote primary cilia formation. Th indicated proteins were overexpressed, in cells incubated in either standard medium (NM) or serum-starvation medium (SSM), and the primary cilia counted. Bar chart of Table 1.

Our data showed that the ODF2 levels are increased in basal bodies compared to those in the centrosomes, and that the ODF2 levels correlated inversely with the CP110 levels. Thus, ODF2 regulated the elimination of the inhibitory cap at the distal end of the mother centriole to allow the cilia to grow out. Therefore, we asked whether the increased expression of ODF2 would stimulate primary cilia formation. The cells were cultured in either standard medium or serum-deprived medium, and the percentage of ciliated cells was determined by decorating for ARL13b. We found an increase from ~30% of ciliated cells in the standard medium to >70% in the serum-deprived medium. However, no significant differences, but rather slight decreases, were observed between the control cells and the cells transfected with either *hCenexinΔGFP*, *hCenexin::GFP*, or *p138NC::mRFP::FKBP*, whether incubated with or without rapamycin (Table 1, Figure 8). We concluded that neither an increase in ODF2 nor incubation with rapamycin is sufficient for primary cilia formation.

### 3.7. Ciliogenesis and Neurl4 Expression

**Figure 9:**
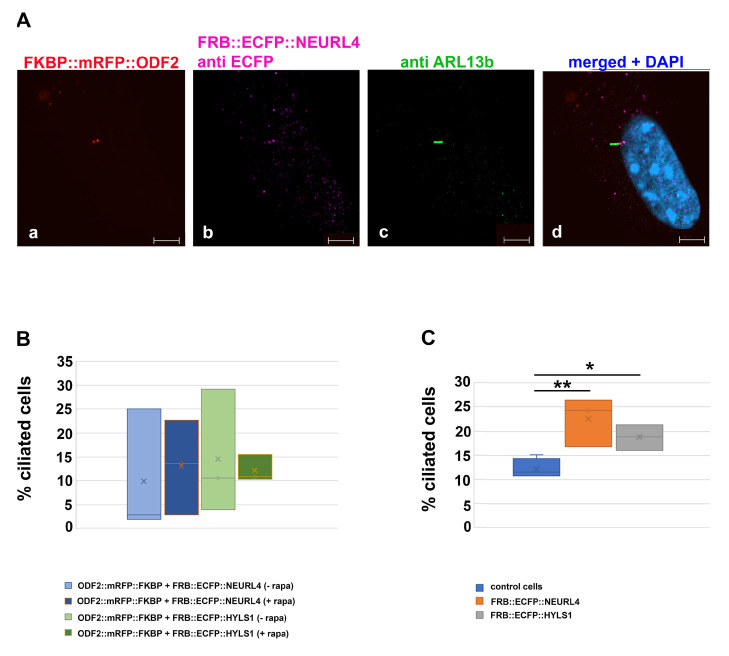
Increased ciliation by overexpression of either FRB::ECFP::NEURL4 or FRB::ECFP::HYLS1. (**A**) Exemplary demonstration of ciliated cells. Cilia were detected by decoration of ARL13b ((**c**), green) and the ECFP-tagged fusion proteins by anti GFP staining ((**b**), pink), in this case, FRB::ECFP::NEURL4. ODF2::mRFP::FKBP (when overexpressed) was detected by its RFP autofluorescence ((**a**), red). Merged image with DAPI staining ((**d**), blue). ODF2-mediated recruitment of NEURL4 was induced by rapamycin treatment. Scale bars: 5 µm. (**B**) Overexpression of ODF2 and NEURL4 or HYLS1 did not induce cilia formation. (**C**) Overexpression of either FRB::ECFP::NEURL4 or FRB::ECFP::HYLS1 caused an increase in ciliated cells in the total cell population compared to the untreated control cells (*p* < 0.05 *, *p* < 0.01 **). Cells were transfected with the indicated constructs, either without (−rapa) or with rapamycin treatment (+rapa), and the primary cilia were counted.

Next, we asked whether the rapamycin-induced dimerization with ODF2, causing the recruitment of NEURL4 to the centriole, promoted cilia formation. The cells were transfected with *p138NC::mRFP::FKBP* and *FRB::ECFP::Neurl4* and either incubated without or with rapamycin, followed by immunodecoration of ARL13b. An average of 12% ciliated cells were found in the untreated control cells (ciliated cells/total cells counted: 54/506, 78/516, 61/519, 57/508, four biological replicates). When transfected with both plasmids, around 10% of transfected cells were ciliated, without rapamycin incubation (transfected cells with cilium/total transfected cells counted: 2/102, 26/104, 3/104, three biological replicates), and rapamycin-induced dimerization caused an increase to 13% (transfected cells with a cilium/total transfected cells counted: 25/110, 14/103, 3/103, three biological replicates). For cilia counting, only transfected cells that were identified by the autofluorescence of the 138NC::mRFP::FKBP-protein were considered (Figure 9A,B). When FRB::ECFP::NEURL4 was expressed, we found an increase in ciliated cells in the total cell population to approximately 23% (ciliated cells/total cells counted: 135/509, 84/501, 122/501, three biological replicates), which is significant according to the Student’s *t*-test (two-sided, homoscedastic, *p* = 0.012588 *) (Figure 9C).

### 3.8. Formation of Higher Order Structures by Interaction between HYLS1 and ODF2

Finally, we investigated the effect of HYLS1 on ciliogenesis. The coding sequence of *Hyls1* was cloned in-frame into p*FRB::ECFP*, yielding plasmid p*FRB::ECFP::Hyls1*. The expression of FRB::ECFP::HYLS1 in NIH3T3 cells caused an increase in the ciliated cells to approximately 19% (ciliated cells/total cells counted: 82/510, 97/515, 108/505, three biological replicates), compared to approximately 12% in the untreated control cells (see above), which is statistically significant, according to the Student’s *t*-test (two-tailed, homoscedastic, *p* = 0.01356664 *) (Figure 9C). Expression of both 138NC::mRFP::FKBP and FRB::ECFP::HYLS1 caused ciliation in approximately 14.5% of the transfected cells (ciliated transfected cells/total transfected cells counted: 11/104, 4/102, 30/103, three biological replicates), and in approximately 13% of the transfected cells when dimerization was induced with rapamycin (ciliated transfected cells/total transfected cells counted: 11/107, 16/103, 11/102, three biological replicates) (Figure 9B). The one-way ANOVA test showed no significant differences when comparing the transfected ciliated cells within the total transfected cells, nor when comparing them with the control cells (Figure 9B). Thus, neither the overexpression of ODF2/138NC and NEURL4 or HYLS1 nor the recruitment to the centrosome induced cilia formation. When comparing ciliation in the total cell population, the one-way ANOVA revealed significant differences compared to the control cells (Figure 9C). Post-hoc tests (Tukey, Scheffé, Holm–Bonferroni, *p* < 0.01 ** by Holm–Bonferroni pairwise comparison) identified significant differences due to the overexpression of NEURL4. The overexpression of HYLS1 is significant different by Holm–Bonferroni pairwise comparison (*p* < 0.05 *).

**Figure 10:**
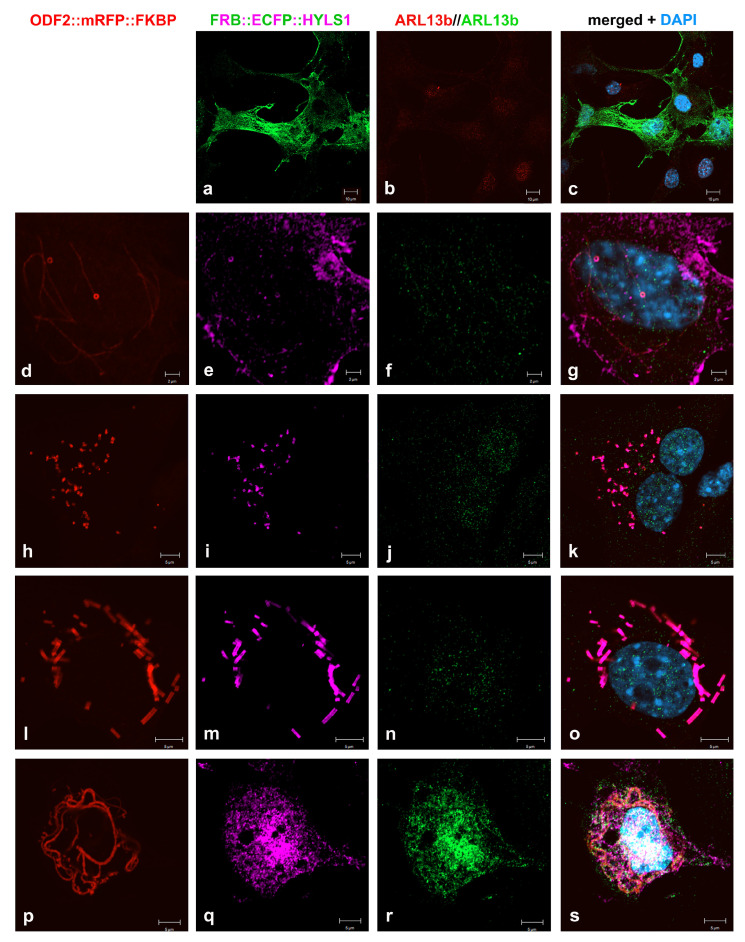
Formation of tubes and rings by interaction of ODF2 and HYLS1. (**a**–**c**) Cytoplasmic expression of FRB::ECFP::HYLS1. Anti-GFP immunostaining for the detection of FRB::ECFP::HYLS1 (green) and ARL13b (red). (**d**–**g**,**h**–**k**,**l**–**o**) Formation of higher-order structures, i.e., tubes and rings, by co-expression and co-localization of 138NC::mRFP::FKBP and FRB::ECFP::HYLS1. Autofluorescence of mRFP-tagged 138NC (red), and anti-GFP immunodecoration of FRB::ECFP::HYLS1 (pink) and ARL13b (green). (**p**–**s**) Formation of fibers by 138NC::mRFP::FKBP (red autofluorescence) without co-localization of FRB::ECFP::HYLS1 (pink). Anti-GFP immunostaining of FRB::ECFP::HYLS1 (pink) and ARL13b (green). Localization of 138NC::mRFP::FKBP (**d**,**h**,**l**,**p**), FRB::ECFP::HYLS1 (**a**,**e**,**i**,**m**,**q**), ARL13b (**b**,**f**,**j**,**n**,**r**), and merged images including nuclear staining with DAPI (blue) (**c**,**g**,**k**,**o**,**s**). Bars are 10 µm (**a**–**c**), 2 µm (**d**–**g**), and 5 µm (**h**–**s**).

FRB::ECFP::HYLS1 showed cytoplasmic expression in NIH3T3 cells (Figure 10a). The co-expression of both 138NC::mRFP::FKBP and FRB::ECFP::HYLS1 indicated protein interaction. Strikingly, the formation of distinct ring- and tube-like structures were found, staining positive for both the mRFP-autofluorescence of 138NC::mRFP::FKBP and the immune-decoration of HYLS1, mediated by incubation with anti-GFP antibodies (Figure 10d–g,h–k,l–o). The tubes and rings had a diameter of approximately 560 nm (calculated from 23 measurements taken from Figure 10d,i). Higher-order structures were found in some of the transfected cells, while in some cells, even the colocalization to 138NC::mRFP::FKBP fibers could not be detected (Figure 10p–s). The formation of tube-like structures and rings, which are most likely tubes in cross-section, was independent of rapamycin-induced dimerization, suggesting that ODF2 and HYLS1 interact with each other to promote the formation of higher-order structures.

## 4. Discussion

### 4.1. Rapamycin-Induced Targeting to the Centriole

The rapamycin-induced dimerization system has been widely used for the targeted recruitment of proteins [58]. Here, we asked whether chemically induced dimerization can be used to control the location of centriolar proteins to modify the composition and function of centrioles. Specifically, we investigated whether the formation of primary cilia can be induced by the targeted recruitment of proteins that are essential for ciliary growth. We have chosen ODF2 as the binding partner for the recruitment of NEURL4 and HYLS1 because ODF2 is intrinsically located in the mother centriole and the basal body. NEURL4 and HYLS1 are essential for the elimination of the inhibitory cap, consisting of CP110, CEP97, CEP290, and other proteins, that is present at the distal ends of mature centrioles/basal bodies and preventing axoneme extension [34]. We have fused the FK506 binding protein FKBP12 to ODF2 and the FRB-domain to either NEURL4 or HYLS1 and induced dimerization by rapamycin treatment. We show here the efficient recruitment of NEURL4 to the centriole by dimerization with ODF2 after mild rapamycin treatment as proof of principle.

### 4.2. ODF2 Controls CP110 Levels

ODF2 is mandatory for the formation of primary cilia and facilitates the docking of ciliary vesicles to distal appendages (DAs) and the formation of the ciliary membrane via interaction with the GTPase membrane trafficking regulator Rab8a [30,56,57]. Furthermore, the assembly of subdistal appendages (SDAs) depends on ODF2, which is located near the centriole wall. The formation of the primary cilium begins at the distal end of the mother centriole with the docking of ciliary vesicles to the tip of DAs and the concurrent extension of the ciliary axoneme [31]. DAs and SDAs are assembled by the sequential recruitment and positioning of specific proteins when the daughter centriole is converted into the mature or mother centriole [26,27]. DAs are assembled by the orchestrated recruitment of CEP90, CCDC41/CEP83, CEP123/CEP89, SCLT1, CEP164, and FBF1, i.a., and assembly crucially depends on C2CD3, localized at the distal ends of the centrioles [63,64,65,66,67,68]. CEP164 is positioned at the tip of the DAs and initiates cilium formation by the docking of ciliary vesicles [29]. However, an essential requirement for centriole maturation and appendage formation is the removal of DCPs (daughter centriole enriched proteins), a specific set of proteins including CEP120, centrobin, and NEURL4 that are recruited to the nascent daughter centriole [23,24,25,69]. DCP removal is regulated by TALPID3, located at the distal ends of both centrioles, and C2CD3, and TALPID3-deficiency abrogates DCP removal, the assembly of DAs, and ciliogenesis, without affecting SDA assembly [57].

In addition, the inhibitory cap at the distal ends of the centrioles consisting of CP110 and other proteins must be removed, which is achieved by degrading CP110 [16,32,34,35,70]. CP110 is mainly degraded by the ubiquitin-dependent proteasome pathway in which the binding to the NEURL4-HERC2 complex plays an essential role [39,40,42,71]. Targeting NEURL4 to the centrosome is sufficient to remove CP110 and restore ciliogenesis [41]. The transient translocation of NEURL4 to the mother centriole early during ciliogenesis requires the physical proximity between the mother and the daughter centrioles [41]. In summary, NEURL4 appears to play a role in both centriole maturation and axoneme extension. The removal of DCPs, including NEURL4, is the initial step in centriole maturation and appendage formation, while NEURL4 appears to be necessary for the degradation of the inhibitory cap at the distal end to allow the cilium to form. NEURL4, therefore, must be recruited to the distal end of the mother centriole to promote CP110 degradation.

We have demonstrated the efficient recruitment of NEURL4 to the centriole by rapamycin-induced dimerization with ODF2. However, we found no indications that the targeted recruitment of NEURL4 to the distal end of the mature centriole promoted the loss of CP110. Although we found a significant decrease in CP110 at the basal body in ODF2 and NEURL4-transfected cells, the loss of CP110 was independent of rapamycin treatment. These results suggested that ODF2 may play an essential role in the elimination of CP110. To further substantiate this finding, we depleted ODF2 by transfection of the short hairpin-plasmid *sh3* or *Odf2* siRNA and measured the amount of CP110 at the centrosome. We found an opposing trend, in that a decrease in ODF2 correlated with an increase in CP110, and vice versa, in the rescue experiments. Our data thus show, for the first time, that ODF2 is essential for the elimination of CP110, not only in the basal bodies after induction of cilium formation, but also in the centrosomes. In retrospect, ODF2 is an inappropriate dimerization partner for NEURL4, if the effect of NEURL4 on cilia formation is to be studied.

The importance of NEURL4 for the degradation of CP110 is well documented [41]. Furthermore, the observation that the master regulator of the ubiquitin proteasome system, the deubiquitinase ubiquitin-specific protease-14 (UPS14), controls cilia formation and that the generation of linear ubiquitin chains at CP110 by the linear ubiquitin chain assembly complex (LUBAC) is required for CP110 removal and ciliogenesis, highlights the importance of the ubiquitin-dependent proteolysis of CP110 [72,73]. We found that ectopically expressed NEURL4 was localized to the centrioles without rapamycin treatment, suggesting the intrinsic targeting of NEURL4 to the centrioles, but that rapamycin-mediated dimerization with ODF2 caused a large increase in the concentration of NEURL4 at the centrioles. Moreover, our data suggest that a specific amount of NEURL4 is sufficient to induce CP110 removal, and that its further recruitment has no additional effect on CP110 degradation.

Although NEURL4 seems to play a major role in the ubiquitin-dependent proteolysis of CP110, it is unclear how NEURL4 is recruited, activated, or stabilized at the basal body prior to the extension of the ciliary axoneme. Our data indicated an involvement of ODF2 in this process. We have shown that ODF2 plays an essential role in controlling the amount of CP110 at the centriole/basal body. This is further supported by the significant enrichment of ODF2 in the basal bodies over the centrosomes in both proliferating and serum-starved cells, and by the observation that a higher amount of ODF2 in the basal body correlated with a lower amount of CP110, and vice versa, in the attached daughter centriole. ODF2 is a structural protein without any known enzymatic activity. Secondary structure predictions indicate that ODF2 is a protein composed mainly of alpha-helix structures, which can form coiled coils [74,75,76]. Thus, ODF2, like many coiled-coil proteins, most likely acts in nucleating and scaffolding large macromolecular complexes [77]. More than 150 proteins interacting with ODF2 have been annotated in BioGRID4.4, including NEURL4 and CP110, both identified by the proximity label-MS [78,79]. Our data show that the amounts of ODF2 and CP110 are interdependent, suggesting that ODF2 most likely acts as a scaffold for NEURL4 binding, which in turn promotes the degradation of CP110.

### 4.3. ODF2 Is Not Sufficient to Promote Cilia Formation

We then asked whether the overexpression of ODF2 might promote cilia formation via the removal of CP110. One of three different plasmids encoding ODF2 was transfected, and the percentage of ciliated cells was determined. We found that the percentage of ciliated cells was in the same order of magnitude as in the non-transfected control cells. Therefore, we concluded that the overexpression of ODF2 is not sufficient to promote cilia formation. We also found no significant increase in ciliated cells when ODF2 (*p138NC::mRFP::FKBP*) and NEURL4 (*FRB::ECFP::Neurl4*) were co-transfected and treated either without or with rapamycin. The percentage of ciliated cells was similar to that of the non-transfected control cells. However, the overexpression of only NEURL4 resulted in a significant increase in ciliated cells, suggesting that increased levels of NEURL4 may increase the degradation of CP110. CP110 has a dual function as a suppressor and promoter of ciliogenesis, as it is also required for SDA assembly and cilia formation in vivo, suggesting that optimal cellular CP110 levels must be strictly balanced [36,37,38]. We, therefore, hypothesize that a moderate increase in NEURL4 would promote the degradation of CP110 to allow axoneme extension and cilium formation, whereas in the presence of excess ODF2, the amount of CP110 may have fallen below a certain threshold, preventing cilium formation. The degradation of CP110 itself is not sufficient for cilium formation. In the G2-phase, in which only a few cells have a primary cilium, CP110 interacts with Cyclin F, which is the substrate-recognition subunit of the SCF ubiquitin ligase complex, on centrioles, causing CP110 ubiquitylation and degradation by the proteasome [39]. In addition, other CP110 binding partners are involved in the regulation of cilium assembly [32]. The calcium-binding protein centrin-2 (CETN2), an interactor of CP110, is essential for the efficient removal of CP110 and primary ciliogenesis upon serum starvation [80]. Centrobin (CNTROB) is a component of the DCPs, enriched at the daughter centrioles, but it also associates with the mother centriole upon serum deprivation and is necessary for primary ciliogenesis [81]. Additionally, axoneme extension requires the microtubule (MT)-associated protein/MT affinity regulating kinase 4 (MARK4) [51]. MARK4 locates at the basal body and the ciliary axoneme. The depletion of MARK4 blocked ciliogenesis and prevented the exclusion of CP110/CEP97 from the mother centriole. MARK4 is activated by LKB1/STK11, recruiting ODF2 to the centriole and phosphorylating it. However, the effect of MARK4 on CP110 and cilia formation could be a purely indirect effect, as it primarily affects the recruitment of ODF2, which in turn promotes CP110 removal and cilia formation.

### 4.4. Co-Localization of ODF2 and HYLS1

The disappearance of CP110 from the basal body coincides with an enrichment of the serine/threonine kinase TTBK2, a microtubule plus-end tracking protein that phosphorylates not only tau and tubulin, but also CEP164, CEP97, and MPP9 [46,82,83]. TTBK2 is recruited to the centriole by CEP164, which is required for the phosphorylation of CEP83 and the subsequent removal of CP110 and initiation of ciliogenesis [43,44,45,46]. The binding of TTBK2 to CEP164 is regulated by phosphatidylinositol-4-phosphate (PtdIns(4)P) levels at the centrosome/ciliary base [47]. Upon serum starvation, the phosphatidylinositol 5-phosphatase (INPP5E) departs from the centrosome, causing depletion of PtdIns(4)P by the activity of phosphatidylinositol(4)P 5-kinase (PIPKIγ), thus freeing CEP164 for the binding with TTBK2 [48]. Furthermore, PIPKIγ recruits HYLS1 (hydrolethalus syndrome protein 1) to the ciliary base, which in turn causes the activation of PIPKIγ, the depletion of centrosomal PI(4)P, and axoneme extension [50]. HYLS1 has been shown to play an essential role in cilia formation [49]. Given the central role of HYLS1 in regulating PtdIns(4)P-levels at the ciliary base, the recruitment of TTBK2, and the removal of CP110, we asked here whether chemically induced recruitment of HYLS1 to the centriole via dimerization with ODF2 promotes cilia formation. When ODF2 (*p138NC::mRFP::FKBP*) and HYLS1 (*FRB::ECFP::Hyls1*) were co-expressed in NIH3T3 cells, the percentage of ciliated cells was in the same order of magnitude as in the non-transfected control cells, even when dimerization was induced by rapamycin treatment. However, we observed a statistically significant increase in ciliation (p*) when HYLS1 was overexpressed, an observation also made for NEURL4-overexpression (see above). We, therefore, speculate that, similar to the case of NEURL4, a moderate increase in HYLS1 promotes cilia formation by increasing the removal of CP110, but that a further increase in HYLS1 at the centriole by targeted recruitment via ODF2 prevents cilia formation because CP110 has fallen below a certain threshold level. We observed the co-localization of ODF2 and HYLS1 in the cytoplasm of transfected cells and their association into tubes and rings. Since tubes and rings were also observed in cells without rapamycin-induced dimerization, ODF2 most likely interacts with HYLS1 to promote the formation or stabilization of higher-order structures. The observed tubes and rings have an average diameter of approximately 560 nm and are therefore most likely not centrioles, which have a diameter of only ~250 nm in vertebrate cells [84]. The width of these structures is similar to the width of vertebrate sperm tails, which ranges from >640 to ~1000 nm, depending on the species and the section of the sperm tail [85,86]. HYLS1 is highly expressed in testis (GeneCards) and is important for flagella formation in *Drosophila* spermatids [87]. It, therefore, seems possible that the interaction between ODF2 and HYLS1 promotes the formation of accessory structures similar to the outer dense fibers in the sperm tails in the cytoplasm of transfected cells, and that their interaction may also be important for the formation of the outer dense fibers and the stability of the sperm tails.

## 5. Conclusions

We used the rapamycin-induced dimerization system to efficiently recruit proteins to the centriole. We found that ODF2 is essential for the efficient removal of CP110. Since ODF2 is a structural protein implicated in the nucleation and scaffolding of macromolecular complexes, we hypothesize that ODF2 recruits and binds NEURL4 at the centriole, enabling the degradation of CP110. The forced expression of ODF2, with or without concomitant overexpression of its interacting proteins NEURL4 or HYLS1, did not stimulate cilia formation, whereas the overexpression of either NEURL4 or HYLS1 resulted in a significant increase in ciliated cells, suggesting that CP110 levels must be tightly controlled to promote cilia formation. Further analyses are required to clarify the effects of increasing the amount of NEURL4 or HYLS1 at the centrosome on CP110 levels and cilium formation.

## Figures and Tables

**Table 1:** Overexpression of ODF2 is not sufficient for primary cilia formation. Cells were transfected with the indicated plasmids, cultivated in either standard medium or serum-deprived medium, and p138NC::mRFP::FKBP-transfected cells were treated either with or without rapamycin. Primary cilia were decorated for ARL13b, manually counted by focusing through all focal planes, and the percentage of ciliated cells was calculated.

Cultivation Conditions	Plasmid Transfected	Ciliated Cells/Total Cells	% Ciliated Cells	Average %
**Standard medium**	**not transfected** **(control)**	177/507	35	33.5
		182/565	32	
	hCenexinΔGFP	136/514	27	26
		135/539	25	
	hCenexin::GFP	231/507	46	40
		177/523	34	
	138NC::mRFP::FKBP (without rapamycin)	130/505	26	24
		110/502	22	
		127/503	25	
	138NC::mRFP::FKBP (with rapamycin)	220/507	43	31
		153/505	30	
		102/498	21	
Serum-deprived	not transfected (control)	390/511	76	77
		402/503	80	
		392/518	76	
	hCenexinΔGFP	340/505	67	66
		394/512	77	
		282/509	55	
	hCenexin::GFP	360/508	71	71
		374/521	72	
	138NC::mRFP::FKBP (without rapamycin)	379/550	69	
		365/501	73	73
	138NC::mRFP::FKBP (with rapamycin)	400/500	80	74
		405/510	79	
		231/370	62	

## Data Availability

Not applicable.
